# Conserved host-pathogen interactions identify novel treatment options in betacoronavirus infections

**DOI:** 10.1038/s41392-021-00480-z

**Published:** 2021-02-10

**Authors:** Soeren Lukassen, Roland Eils

**Affiliations:** 1 Charité ‐ Universitätsmedizin Berlin, corporate member of Freie Universität Berlin, Humboldt‐Universität zu Berlin, and Berlin Institute of Health, Berlin, Germany; 2 Center for Digital Health, Berlin Institute of Health (BIH), Berlin, Germany; 3 Health Data Science Unit, Heidelberg University Hospital and BioQuant, Heidelberg, Germany

**Keywords:** Infectious diseases, Infectious diseases

In a recent study published in Science,^1^ Gordon et al. investigate the host-pathogen interactome of the coronaviruses SARS-CoV-1, SARS-CoV-2, and MERS-CoV, all of which caused lethal outbreaks in the past two decades. They functionally characterize a number of these interactions, leveraging the information to identify promising candidate molecules for drug-repurposing.

Since 2002, there were three major outbreaks of novel coronaviruses in humans. SARS-CoV-1 emerged in 2002, MERS-CoV in 2013, and SARS-CoV-2 in 2019. While the first two viruses only spread regionally and caused under 1000 deaths each, SARS-CoV-2 has caused almost 55 million cases and over 1.3 million deaths globally as of November 2020. The rapid succession of outbreaks necessitates research into the conserved features of coronaviruses to build upon this knowledge should yet another coronaviral disease emerge. Conserved interactions are also more likely to be essential for virus function and less likely to rapidly evolve to develop drug resistance.

**Fig. 1:**
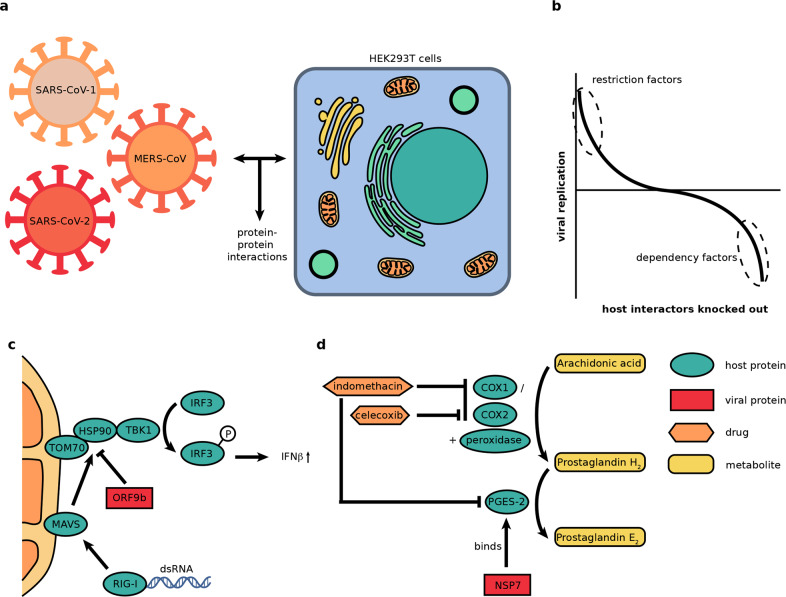
**a**, **b** Interaction candidate selection approach used by Gordon et al. to identify druggable host factors involved in SARS-CoV-2 infection. The authors first screened for interactors with viral proteins of SARS-CoV-1, SARS-CoV-2, and MERS-CoV (**a**) and then filtered these through a knockout/-down screen revealing restriction and dependency factors, respectively (**b**). **c** Proposed mechanism of action for an ORF9b-mediated disruption of viral dsRNA sensing and IFNβ production. **d** Mechanism of action of indomethacin and the control drug celecoxib in prostaglandin synthesis

Gordon et al. complement their existing SARS-CoV-2 host-pathogen protein-protein interaction (PPI) data^2^ with mass-spectrometry data obtained from a screen using tagged SARS-CoV-1 and MERS-CoV proteins as baits (Fig. 1a)^1^. Immunostainings performed for most tagged and some untagged viral proteins found that the subcellular localization of homologous proteins is generally conserved, with some differences likely attributable to the experimental setup. As expected, the overlap of protein targets followed the evolutionary conservation, with SARS-CoV-1 and SARS-CoV-2 being more similar to each other than to MERS-CoV, with functional groups of host interactors being more conserved than the proteins themselves. A number of non-orthologous viral proteins such as SARS-CoV-2 Nsp8 and MERS-CoV Orf4a shared the same interactions, while their orthologs did not. This repurposing suggests that the host factors involved, rather than the viral interactors, may be druggable targets that could not easily be circumvented by viral plasticity.

To identify PPIs relevant to the infection process, A549-ACE2 cells and Caco-2 cells were infected with SARS-CoV-2 after treating them with siRNAs and sgRNAs against the identified interactors and the known entry-receptor *ACE2*, respectively. By measuring viral RNA in the supernatant or titrating the virus on Vero-E6 cells, they identified 31 and 40 dependency factors by siRNA and CRISPR screen, respectively, and three and four viral restriction factors (Fig. 1b). Twelve dependency factors were found to interact with Nsp7 of at least one of the coronaviruses, with PGES-2 interacting with all three. SARS-CoV-specific shared interactions with dependency factors included Orf8-IL17RA, Orf9b-Tom70, and Nsp6-SIGMAR1. The knockdown/-out of *ACE2* almost completely abolished virus production indicating that alternative routes of infection that have been discussed, such as CD147, do not play a role here.

The CryoEM structure of the Orf9b-Tom70 complex shows an interaction via the recognition site for mitochondrial targeting sequences on Tom70, a mitochondrial membrane transporter. In Orf9b-bound Tom70 a crucial residue for the interaction with HSP90 was found shifted compared to the yeast homolog, suggesting a potential loss of this interaction. This would have far-reaching consequences, as this interaction activates TBK1/IRF3, which is required for intracellular virus sensing through RIG-I signaling and subsequent IFN-β production (Fig. 1c). A recent study^3^ found 3.5% of patients with life-threatening COVID-19 to harbor predicted loss-of-function variants in interferon signaling genes, among them different autosomal dominant mutations in TBK1 and IRF3. Another study^4^ identified autoantibodies against type I interferons in 10% of patients with severe COVID-19 pneumonia, further highlighting the important role of the innate immune system in COVID-19. While injected IFN-β did not appear to have a benefit in the large-scale SOLIDARITY trial, a smaller study performed with inhaled IFN-β increased the odds of clinical improvement in the treatment cohort.^5^

Two further host factors interacting with viral proteins of either the two SARS viruses or all three coronaviruses and aiding its replication, PGES-2 and sigma-1, have known inhibitors that are in clinical use, allowing the study of health records for COVID-19 outcomes in patients treated with these. PGES-2 is inhibited by indomethacin, a non-steroidal anti-inflammatory drug that is also a non-selective COX inhibitor, reducing prostaglandin synthesis through two different modes of action and supporting IFN-γ-induced gene expression (Fig. 1d). No in vitro effect of indomethacin on SARS-CoV-2 replication could be identified. For a clinical evaluation, Gordon and colleagues obtained medical billing records of 738,933 patients diagnosed with COVID-19. Using the selective COX-2 inhibitor celecoxib as control, they calculated the odds of hospitalization of outpatients diagnosed with SARS-CoV-2 within 21 days of starting treatment with either drug. Samples were matched using risk set sampling (RSS) based on demographic and clinical data, with further refinement using propensity score (PS) matching. Although the stringent combination of RSS and PS led to an odds ratio (OR) for hospitalization or inpatient services of 0.33 (95% CI: 0.03–3.19), the filtering based on RSS alone resulted in an OR of 0.25 (0.08–0.76). While this is certainly no conclusive proof of a benefit of indomethacin treatment to prevent hospitalization of COVID-19 patients, it is a compelling finding that warrants further research.

The Sigma-1 receptor is a target of typical, but not atypical antipsychotics. Using an approach similar to that for indomethacin, the OR for new users of typical or atypical antipsychotics hospitalized with COVID-19 to require mechanical ventilation was calculated. While RSS alone yielded an OR of 0.56 (0.31–1.02), the matching based on RSS and PS resulted in an OR of 0.46 (0.23–0.93), demonstrating a potential benefit of typical antipsychotics in preventing the need for mechanical ventilation.

Gordon et al. present an impressive tour de force by combining the generation of a widely useful resource on coronavirus host-pathogen interactions with both mechanistic and clinical insights, leveraging these to provide new avenues in the treatment of COVID-19. These will have to be investigated further to assess their efficacy and safety in a clinical setting, as well as patient populations that could benefit from them. Both antipsychotics and indomethacin can have serious adverse effects. Indomethacin can interfere with hypertensive medication, thus potentially exacerbating this risk factor for COVID-19, and impair kidney function as is also observed in COVID-19. IFN-β on the other hand may only be effective in early stages of the disease as viral clearance is mostly completed at ten days after the onset of symptoms, a time span below the average duration of mechanical ventilation in critical cases.
